# Burden and impact of *Plasmodium vivax* in pregnancy: A multi-centre prospective observational study

**DOI:** 10.1371/journal.pntd.0005606

**Published:** 2017-06-12

**Authors:** Azucena Bardají, Flor Ernestina Martínez-Espinosa, Myriam Arévalo-Herrera, Norma Padilla, Swati Kochar, Maria Ome-Kaius, Camila Bôtto-Menezes, María Eugenia Castellanos, Dhanpat Kumar Kochar, Sanjay Kumar Kochar, Inoni Betuela, Ivo Mueller, Stephen Rogerson, Chetan Chitnis, Dhiraj Hans, Michela Menegon, Carlo Severini, Hernando del Portillo, Carlota Dobaño, Alfredo Mayor, Jaume Ordi, Mireia Piqueras, Sergi Sanz, Mats Wahlgren, Laurence Slutsker, Meghna Desai, Clara Menéndez, on behalf of the PregVax Study Group

**Affiliations:** 1 Barcelona Center for International Health Research, CRESIB, ISGlobal, Hospital Clínic - Universitat de Barcelona, Barcelona, Spain; 2 Gerência de Malária, Fundação de Medicina Tropical Dr. Heitor Vieira Dourado, Manaus, Brazil; 3 Instituto Leônidas e Maria Deane, FIOCRUZ Amazônia, Manaus, Brazil; 4 Caucaseco Scientific Research Center/Universidad del Valle, Cali, Colombia; 5 Centro de Estudios en Salud, Universidad del Valle de Guatemala, Guatemala, Guatemala; 6 Department of Medicine, Sardar Patel Medical College, Bikaner, Rajasthan, India; 7 Papua New Guinea Institute of Medical Research, Madang, Madang Province, Papua New Guinea; 8 The Walter and Eliza Hall Institute, Parkville, Victoria, Australia; 9 Department of Medicine (Royal Melbourne Hospital), The University of Melbourne, Parkville, Victoria, Australia; 10 International Center for Genetic Engineering and Biotechnology, New Delhi, India; 11 Department of Infectious, Parasitic and Immunomediated Diseases, Istituto Superiore di Sanità, Rome, Italy; 12 Institució Catalana de Recerca i Estudis Avançats, Barcelona, Spain; 13 Karolinska Institute, Stockholm, Sweden; 14 Malaria Branch, Division of Parasitic Diseases and Malaria, Center for Global Health, Centers for Disease Control and Prevention, Atlanta, Georgia, United States of America; Johns Hopkins Bloomberg School of Public Health, UNITED STATES

## Abstract

**Background:**

Despite that over 90 million pregnancies are at risk of *Plasmodium vivax* infection annually, little is known about the epidemiology and impact of the infection in pregnancy.

**Methodology and principal findings:**

We undertook a health facility-based prospective observational study in pregnant women from Guatemala (GT), Colombia (CO), Brazil (BR), India (IN) and Papua New Guinea PNG). Malaria and anemia were determined during pregnancy and fetal outcomes assessed at delivery. A total of 9388 women were enrolled at antennal care (ANC), of whom 53% (4957) were followed until delivery. Prevalence of *P*. *vivax* monoinfection in maternal blood at delivery was 0.4% (20/4461) by microscopy [GT 0.1%, CO 0.5%, BR 0.1%, IN 0.2%, PNG 1.2%] and 7% (104/1488) by PCR. *P*. *falciparum* monoinfection was found in 0.5% (22/4463) of women by microscopy [GT 0%, CO 0.5%, BR 0%, IN 0%, PNG 2%]. *P*. *vivax* infection was observed in 0.4% (14/3725) of placentas examined by microscopy and in 3.7% (19/508) by PCR. *P*. *vivax* in newborn blood was detected in 0.02% (1/4302) of samples examined by microscopy [in cord blood; 0.05% (2/4040) by microscopy, and 2.6% (13/497) by PCR]. Clinical *P*. *vivax* infection was associated with increased risk of maternal anemia (Odds Ratio-OR, 5.48, [95% CI 1.83–16.41]; p = 0.009), while submicroscopic vivax infection was not associated with increased risk of moderate-severe anemia (Hb<8g/dL) (OR, 1.16, [95% CI 0.52–2.59]; p = 0.717), or low birth weight (<2500g) (OR, 0.52, [95% CI, 0.23–1.16]; p = 0.110).

**Conclusions:**

In this multicenter study, the prevalence of *P*. *vivax* infection in pregnancy by microscopy was overall low across all endemic study sites; however, molecular methods revealed a significant number of submicroscopic infections. Clinical vivax infection in pregnancy was associated with maternal anemia, which may be deleterious for infant’s health. These results may help to guide maternal health programs in settings where *vivax* malaria is endemic; they also highlight the need of addressing a vulnerable population such as pregnant women while embracing malaria elimination in endemic countries.

## Author summary

More than 90 million pregnancies are exposed to *P*. *vivax* infection every year. While it is well known that pregnant women have an increased risk of *P*. *falciparum* infection and disease, much less is known on the epidemiology and the impact of *P*. *vivax* in pregnancy. A health-facility based observational study was conducted in pregnant women living in five vivax endemic countries aimed to determine the burden of the infection in pregnancy and its impact on the mother and the newborn health. We found that the prevalence of *P*. *vivax* malaria in pregnant women attending the routine antenatal clinic visits was overall low across all sites, however submicroscopic infections were unexpectedly high in some areas. Pregnant women with clinical malaria experienced an increased risk of anemia, which may have a deleterious impact on infant health. These findings may be useful for guiding maternal health programs in vivax endemic settings, as well as for malaria elimination activities.

## Introduction

More than a third of the world′s population is at risk of *Plasmodium vivax* infection, 90% of whom live in the Asia and the Pacific regions, while the rest live in the American (6%) and African (3%) regions. [1–3] In comparison with *P*. *falciparum*, *P*. *vivax* infection has received little attention mostly because it has been considered a relatively benign disease. [3, 4] However, in recent years due to the increasing evidence of the severity of *P*. *vivax* infection and the recognition of its economic impact in endemic countries, a greater interest has been devoted to this neglected and complex species. [3, 5] This renewed interest is also enhanced by the recognition that with intensified control efforts *P*. *vivax* has become the predominant malaria species and the major challenge to malaria elimination outside Africa.

It is well recognized that pregnant women have an increased risk of *P*. *falciparum* infection and disease, and its effects on maternal and infant health have been well documented in sub-Saharan Africa. [6] Less is known about the burden of *P*. *vivax* in pregnancy and its impact on maternal and infant health. In 2007, it was estimated that the number of pregnancies at risk of *P*. *vivax* was at least 92 million globally. [7] Some studies on the effect of *P*. *vivax* infection in pregnancy have suggested that the infection is associated with maternal anemia, miscarriage, congenital malaria and preterm delivery, as well as with severe disease. [8–17] These studies were mostly done in Asia, and with some exceptions,[12, 18–20] were based on limited numbers or case reports, thus providing an incomplete picture of the overall burden and impact of *P*. *vivax* in pregnancy. In particular, there were limited data from Latin America.

A comprehensive description of the burden and impact of *P*. *vivax* infection in pregnancy across different malaria transmission settings is needed to guide control strategies. We present here the results of a prospective observational study in pregnant women exposed to *vivax* malaria that aimed to determine the burden of *P*. *vivax* malaria and its impact on pregnancy outcomes. The study followed a multicenter approach including different epidemiological settings broadly representing of *P*. *vivax* endemic areas; one Asian country, one country from the Pacific region and three countries from the Americas. The objectives of the study were to estimate the prevalence of *P*. *vivax* infection at different time points overall and for each of the sites, and the incidence of *P*. *vivax* malaria during pregnancy. It also aimed to assess the clinical impact of *P*. *vivax* infection during pregnancy on maternal health and fetal outcomes.

## Methods

### Ethics

The study protocol was reviewed and approved by the national and/or local ethics review boards in each of the study sites, the Institutional Review Board (IRB) of the Centers for Disease Control and Prevention (CDC), and the Hospital Clinic of Barcelona Ethics Review Committee. The study was conducted in accordance with the Good Clinical Practice Guidelines, the Declaration of Helsinki, and local rules and regulations of each partner country.

### Study design and study areas

This was a health facility-based prospective observational study of pregnant women attending routine antenatal care (ANC) clinics undertaken between June 2008 and October 2011 in five *P*. *vivax* endemic countries—Colombia (CO), Guatemala (GT), Brazil (BR), India (IN) and Papua New Guinea (PNG)–of different malaria endemicity characteristics (Table 1). The historic levels of malaria transmission varied across study sites from hypoendemic in Guatemala, India and Colombia, mesoendemic in Brazil, to hyperendemic in PNG. *P*. *vivax* was the predominant species in all sites except for PNG where *P*. *vivax* co-existed with *P*. *falciparum* (50%) and other species (*P*. *malariae* and *P*. *ovale*, 5%).

**Table 1:** Characteristics of the study sites.

Country	Colombia	Guatemala	Brazil	India	Papua New Guinea
**Sites**	Tierralta, Córdoba	Fray Bartolomé de las Casas, Alta Verapaz	Manaus, Amazonas	Bikaner, Rajasthan	Madang
**Location**	Rural	Rural	Peri-urban	Peri-urban	Peri-urban & Rural
**Malaria transmission**	Hypoendemic	Hypoendemic	Mesoendemic	Hypoendemic	Hyperendemic
**Endemicity**	Perennial transmission	Perennial transmission, peak Apr-Nov	Perennial transmission, peak Jul-Aug	Seasonal transmission, Sep-Dec, Mar-Jun	Perennial transmission
**Plasmodium species**	70% *P*.*vivax*, 30% *P*.*falciparum*	100% *P*.*vivax* ^a^	86% *P*.*vivax*, 14% *P*.*falciparum*^b^	97% *P*.*vivax*, 3% *P*.*falciparum*	45% *P*.*vivax*, 50% *P*.*falciparum*, 5% *P*.*ovale/malariae*
**Vector**	*Anopheles nuneztovari*, *An*.*darlingi*	*Anopheles albimanus*, *An*.*darlingi*, *An*.*vestitipennis*	*An*. *darlingi*	*An*.*subpictus*, *An*.*stephensi*, *An*.*culicifacies*, *An*.*annularis*	*An*.*punctulatus*, *An*.*koliensis*, *An*.*faranti*
**Coverage ANC attendance** (at least 4 ANC visits)	80%	80%	80%	40%	55%^c^
**ITNs Coverage (ownership)**	40%	90%	7%	No ITNs	75%
**Guidelines for treatment of *P*.*vivax* uncomplicated malaria in pregnancy**	CQ full course^d^ + CQ weekly until delivery + PQ 14d 6m post-partum	CQ full course + CQ weekly until delivery + PQ 14d post-partum	CQ full course + CQ weekly 12w + PQ 14d 6m post-partum	CQ full course	CQ full course + SP full dose^e^
**Guidelines for treatment of *P*.*falciparum* uncomplicated malaria in pregnancy**	1st trimester: QNN + CLIN 7d^f^ 2nd-3rd trimesters: AL^g^	CQ full course + PQ 3d post-partum	1st trimester: QNN 3d + CLIN 5d 2^nd-^3^rd^ trimesters: AL	1st trimester: QNN 2nd-3rd trimesters: ACTs	CQ full course + SP full dose^h^
**Guidelines for prevention of malaria in pregnancy**	ADI at each ANC visits	PCD + ITNs^i^	ADI at each ANC visits	PCD	(CQ full course + SP) at 1^st^ ANC visit + CQ weekly until delivery + PCD

Abbreviations: CQ: Chloroquine, PQ: Primaquine, SP: Sulfadoxine-pyrimethamine, QNN: Quinine, CLIN: Clindomycin, AL: Artemeter-Lumefantrine, ITNs: Insecticide-treated bednets, ADI: Active Detection of Infection, ACTs: Artemisin-combined therapy, PCD: Passive detection of cases.

^a^ No P. falciparum cases reported in the area since 2009.

^b^ Data from 2009.

^c^ 79% of women completed at least 1 ANC visit.

^d^ 150mg CQ base (tab 250 mg), 4 tab 1st day and 3 tab 2nd and 3rd day.

^e^ 500mg sulfadoxine/25mg pyrimethamine (3tab, monodosis).

^f^ QNN: 10mg/kg every 8h, CLIN: 10mg/kg every 12h.

^g^ AL: Artemeter (20mg)-Lumefantrine (120mg), twice per day over 3 days.

^h^ New protocol adopted in 2009. Uncomplicated P.falciparum malaria: AL. Uncomplicated P.vivax malaria: AL+PQ.

^i^ Indoor residual spraying and larvicides in general population.

Source of data: Colombia: Montoya-Lerma J, Solarte YA, Giraldo-Calderón GI, Quiñones ML, Ruiz-López F, Wilkerson RC, González. Malaria vector species in Colombia: a review. Mem Inst Oswaldo Cruz 2011, 106:223–238. Chaparro P, Padilla J, Vallejo AF, Herrera S: Characterization of a malaria outbreak in Colombia in 2010. Malar J 2013, 12:330. Guía de atención médica para el diagnóstico y tratamiento de la malaria. Ministerio de Salud de Colombia. 2010. Guatemala: Padilla N, P. Molina, J. Juarez, D. Brown and C.Cordon-Rosales. Potential malaria vectors in northern Guatemala (Vectores potenciales de malaria in la region norte de Guatemala). J Am MosqControl Assoc 1992;8:307–8. Brazil: http://portalweb04.saude.gov.br/sivep_malaria/default.asp. Accessed 15 January 2017. India: Kochar DK, Sirohi P, Kochar SK, Budania MP, Lakhotia JP. Dynamics of malaria in Bikaner, Rajasthan, India (1975–2006). J Vector Borne Dis. 2007 Dec;44(4):281–4. PNG: Papua New Guinea National Department of Health: National Malaria Treatment Protocol. Port Moresby: National Department of Health ed., 1st edition; 2009. Marfurt J, Müeller I, Sie A, Maku P, Goroti M, Reeder JC, Beck HP, Genton B. Low efficacy of amodiaquine or chloroquine plus sulfadoxine-pyrimethamine against Plasmodium falciparum and P. vivax malaria in Papua New Guinea. Am J Trop Med Hyg. 2007 Nov;77(5):947–54. Barnadas C, Koepfli C, Karunajeewa HA, Siba PM, Davis TM, Mueller I. Characterization of treatment failure in efficacy trials of drugs against Plasmodium vivax by genotyping neutral and drug resistance-associated markers. Antimicrob Agents Chemother. 2011 Sep;55(9):4479–81.

### Study procedures

Unselected pregnant women of any age, gestational age, and parity, attending the ANC clinic at each study site, independently of parasitological or disease status, were invited to participate, and after signing a written informed consent were recruited into the study. There were four study visits: recruitment visit, coinciding with an ANC visit; two subsequent scheduled ANC visits one month apart; and at delivery. Project personnel were trained on study procedures that were standardized across all study sites. At each study visit and regardless of the presence of symptoms suggestive of malaria, a capillary blood sample was collected to prepare two thick and thin blood smears and two filter papers (Whatmann 3MM) to determine *Plasmodium* parasitemia. From the same samples the hemoglobin (Hb) concentration was measured at enrolment, at scheduled ANC study visits, at delivery and at any other time the woman was suspected to have malaria. In addition, demographic, obstetric and clinical information were recorded on standardized questionnaires. Pregnant women were encouraged to deliver at the study health facility. In case of home delivery, they were advised to come to the study health facility within the first week of giving birth.

A passive surveillance system to identify study women presenting with clinical malaria was set up at each study health facility. If the women reported any signs and/or symptoms suggestive of clinical malaria, a capillary blood sample was collected to prepare two thick and thin blood smears and two filter papers to determine *Plasmodium* parasitemia, and for determination of Hb concentration.

At delivery, the pregnancy outcome and the clinical information on the mother and the neonate were collected. Placental blood from all women delivering at the health facility was collected onto filter paper for PCR molecular analysis, and two impression smears were stained with Giemsa and read following standard procedures for parasitemia determination. [21] For the preparation of the impression smears a 2.5x2.5 cm^3^ sample from the placenta, that should include the full thickness of the tissue from the maternal to the fetal side, was cut and put in contact with the slide after being dried with a piece of filter paper. In a randomly selected subsample of women (10%) a placental biopsy was collected, following a similar procedure (2.5x2.5 cm^3^ sample) to that for the preparation of the impression smear. The biopsy was kept at 4°C in 50 mL of 10% neutral buffer formalin, processed for histological examination, and stained with haematoxylin and eosin as previously described. [22] The histological examination of placental biopsies for malaria infection was performed by local pathologists trained for the purposes of the study (S1 File). Cord blood samples were collected after birth for parasitemia determination. As soon as the umbilical cord was clamped, cut, and separated from the newborn study trained staff collected 5mL cord blood sample taken from the cord with a syringe and needle. Cord blood was used for preparation of two thick and thin blood smears to determine *Plasmodium parasitemia*, and two filter papers to perform PCR molecular analysis. Newborn samples were collected by heel prick after medical assessment was complete and within the first 12 hours of life also for parasitemia determination.

In GT, BR and PNG gestational age was assessed by the Ballard’s method for deliveries that occurred at the health facility within the first 72 hours after birth [23]. In CO and IN gestational age was assessed by ultrasound assessment performed at enrolment. All newborns were weighed on a digital scale, accurate to the nearest gram, within the first 2 hours of life and examined for any clinical abnormalities. For deliveries occurred at home, the study personnel obtained information on the pregnancy outcome through home visits. All women and newborns with malaria infection or anemia were treated according to the national guidelines in each country. Strategies for malaria control in pregnancy differed across study sites. While these relied on active detection of infection with microscopy at each ANC visit in some countries, in other countries passive detection of cases, or weekly prophylaxis with chloroquine (CQ) until delivery were carried out (Table 1).

At enrolment a subsample of 1500 women (300 per study site) were randomly selected for the determination of the prevalence of *P*. *vivax* and *P*. *falciparum* infection by PCR methods. Likewise, a subsample of 1500 women (300 per study site) was randomly selected at delivery for the same purpose. The 1500 women from whom samples were obtained at delivery were different from those selected at enrolment. A total of 500 placental samples (100 per site) and 500 cord blood samples (100 per site) were also analysed following same methodology. A simple random sampling method was used for the selection of women in each country, and for each time point and compartment. The sample size for prevalence by PCR was agreed among study investigators according to preliminary results of the first 100 selected samples in each site that showed a prevalence of *P*. *vivax* monoinfection of 7.5% by PCR, and on the availability of resources for molecular analyses in the study. PCR assays were not performed on newborn blood samples.

To estimate the impact of submicroscopic *P*. *vivax* and *P*. *falciparum* infections on maternal anemia and low birth weight (LBW), a nested case-control study was conducted. All countries contributed to this pooled analysis. A definition for moderate-severe anemia as Hb less than 8g/dL was agreed for the purpose of the analysis. All moderate-severe anemia cases (n = 342), and LBW cases (<2500g) (n = 327) existing across countries for which a blood smear and a filter paper were available, were included in the case control analysis. A total of 414 controls to anemia cases, and a total of 410 controls to LBW cases, were randomly selected. Logistic regression models used to evaluate the impact of *P*.*vivax* submicroscopic infections on anemia and LBW were adjusted by site and *P*. *falciparum* infections. Similarly, for the evaluation of the impact of *P*. *falciparum* submicrocopic infections, models were adjusted by site and *P*. *vivax* infections.

### Laboratory methods

Giemsa-stained thick and thin blood slides were read onsite in all countries following WHO standard quality-controlled procedures to establish parasite presence and density of *Plasmodium* asexual stages. [24] Two independent expert malaria microscopists read all slides and results were registered in two separate forms. Discrepant results (positive vs. negative) were resolved by a third reading done by a different microscopist. A blood slide was declared as negative only when no parasites were found after reading 200 fields. Results were expressed in parasites/μL after counting the number of parasites per 500 white blood cells or reaching 500 parasites; counting was normalized using estimated leukocyte counts of 8000/μL. External validation of a blood slides subsample (100 slides per country) was done at the Hospital Clinic and at the Hospital Sant Joan de Deu, in Barcelona, Spain. Hb was measured by Coulter Counter (except in PNG where it was done by Hemocue, HemoCue, Ltd, Angelhom, Sweden; accuracy of 0.1 g/dL) using 50–100 μL collected in a 0.5mL EDTA tube (microtainer).

Molecular detection of *Plasmodium* species in samples from CO, GT, BR and PNG was performed by Real Time PCR at the Istituto Superiore di Sanità (ISS) in Rome, Italy. Samples from India were analysed, due to local regulatory requirements, at the International Center for Genetic Engineering and Biotechnology (ICGEB), in New Delhi using the same protocol as that at ISS but adapted for the sake of instrument sensitivity (3^rd^ step at amplification was 72°C for 25 sec instead of 72°C for 5 sec). DNA was extracted from whole blood-spot filter paper from maternal peripheral blood collected at ANC, at delivery and during passive case detection, and from placental and cord blood using Purelink Genomic DNA Kit (Invitrogen). *P*. *vivax* and *P*. *falciparum* infections were detected with a LightCycler 480 system (Roche). Species-specific primers and Taqman probes were selected from the sequence of the small 18S rRNA subunit as previously described by Veron *et al* 2009. [25] Briefly, pre-incubation was at 95°C for 10 min; amplification at 95°C for 10 sec, 50°C for 20 sec and 72°C for 5 sec for 50 cycles. All reactions were in duplicate in a final volume of 20 μL. The sensitivity of the PCR assay performed at the ISS for detection of *P*. *falciparum* infections was between 10–100 times higher in comparison to the microscopy. An external validation of the PCR methods used by the ISS and the ICGEB, for detection of *P*. *vivax* and *P*. *falciparum* species, was performed by the Malaria in Pregnancy Consortium (MiPc) (http://www.mip-consortium.org) in a subset of 20 samples. Internal validation between ISS and ICGEB was also done.

### Data management and statistical analysis

A standardised system for data entry, data management, and statistical analysis was established. All clinical and laboratory data were collected using standardised questionnaires. The data collection and management was performed using the OpenClinica open source software, version 2.0. Copyright OpenClinica LLC and collaborators, Waltham, MA, USA, www.OpenClinica.com. All data were double entered by two independent data clerks at each of the sites. There was a specific URL link to access the data entry software. In PNG data were doubled-entered into form-specific databases (FoxPro 9·0, Microsoft, USA). Validation and cleaning were done using the same software, and statistical analysis was performed using Stata 13 (Stata Corporation, College Station, TX, USA). Differences between proportions were compared using the Pearson′s *chi-squared* test or Fisher's exact test depending on type of variables. For continuous variables, Student’s T-tests were used to compare the groups. Incidence rates were calculated as the number of new episodes/person-year at risk using the Poisson distribution in the exposed and unexposed groups, with primigravid women as the comparator group. The impact of *P*. *vivax* infection on maternal and newborn health was determined through a multicenter-pooled analysis. The case control study for submicroscopic infections with and anemia was analyzed using logistic regression models. We adjusted all regression models for possible operational confounding variables such as country and previous malaria episodes. In the analyses of *P*. *falciparum* infections, they were included in the models as being free of *P*. *vivax* (see definitions section). Likewise, in the analyses of *P*. *vivax* infections, they were included as being free of *P*. *falciparum*. Multivariate analyses were performed by a forward-stepwise procedure, using p<0.05 and p>0.10 from the likelihood ratio test, as enter and remove criteria respectively. Results from the estimated models were expressed as OR and 95% CI. Missing values were coded as such and excluded from analysis.

### Definitions

*P*. *vivax* microscopic monoinfection was defined as the presence of asexual *P*. *vivax* parasites of any density and absence of other *Plasmodium* species on the blood smear. *P*. *vivax* clinical malaria episode was defined as the latter plus any signs or symptoms suggestive of malaria (axillary temperature ≥37.5°C or history of fever in the last 24 hours, headache, arthromyalgias, and/or pallor). *P*. *vivax* submicroscopic infection was defined as a PCR that was positive for *P*. *vivax* and negative for *P*. *falciparum*, with a concomitant blood film negative by microscopy. *P*. *falciparum* microscopic monoinfection was defined as the presence of asexual *P*. *falciparum* of any density and absence of other Plasmodium species on the blood smear. *P*. *falciparum* clinical malaria episode was defined as the latter plus any signs or symptoms suggestive of malaria. *P*. *falciparum* submicroscopic infection was defined as a PCR that was positive for *P*. *falciparum* and negative for *P*. *vivax*, with a concomitant blood film negative by microscopy. The duration of any single malaria episode was estimated as 28 days.

Congenital malaria was defined as presence of asexual *Plasmodium* parasites of any species in the cord blood or in the newborn′s peripheral blood at delivery, regardless of clinical symptoms or signs in the neonate. Placental infection was classified according to a previously established definition. [21] Briefly, acute infection was defined as the presence of parasites, with absent or minimal pigment deposition within fibrin or cells within fibrin, chronic infection as the presence of parasites and a significant amount of pigment deposition, and past infection as the presence of pigment with absence of parasites. Prematurity was defined as gestational age < 37 weeks. LBW was defined as birth weight <2500g.

## Results

### Baseline characteristics of study women

**Fig 1:**
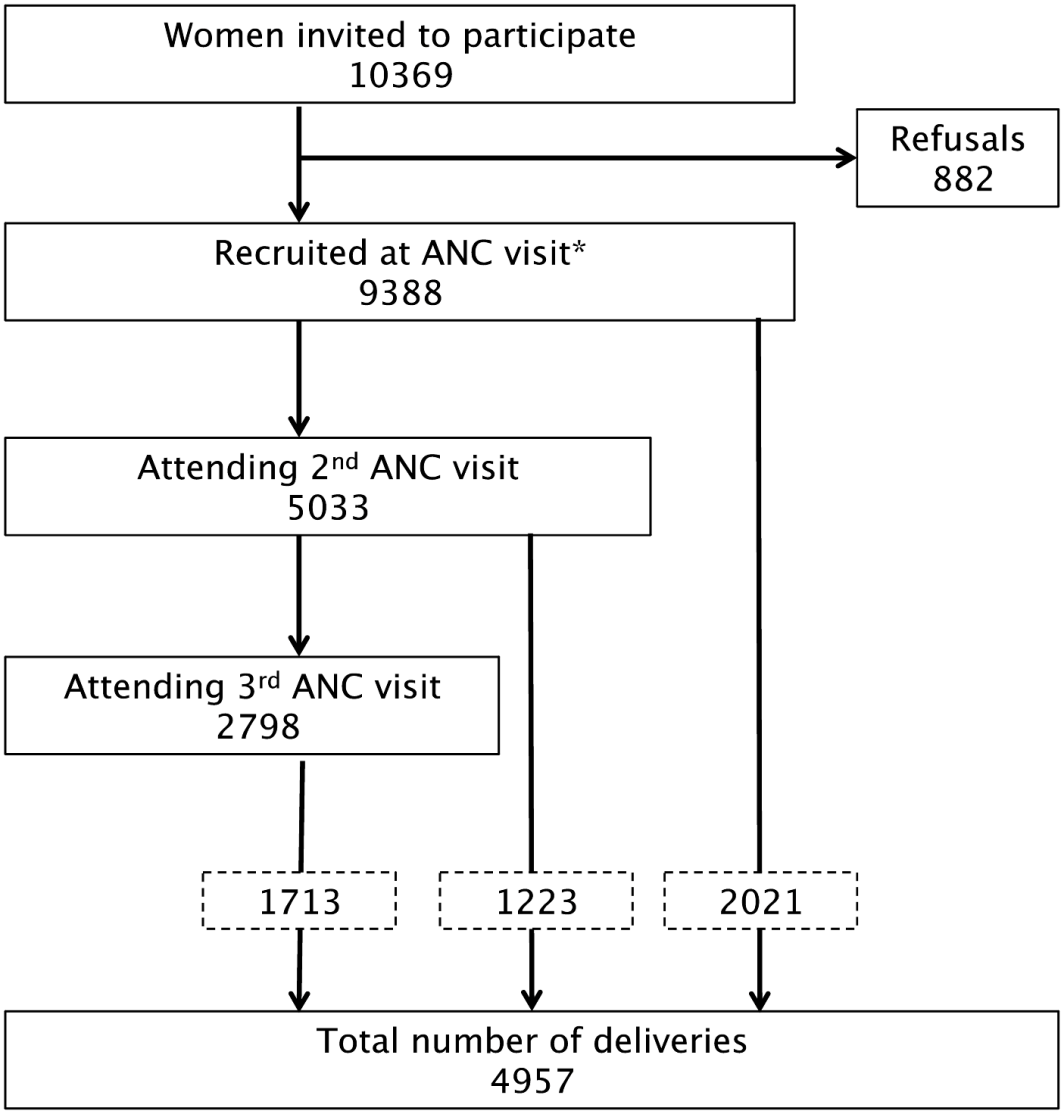
Study profile. * Number recruited at antenatal care per country: 2009 in Guatemala, 2043 in Colombia, 1657 in Brazil, 1982 in India, and 1697 in PNG.

A total of 9388 pregnant women were recruited across all sites (2043 in CO; 2009 in GT; 1657 in BR; 1982 in IN; 1697 in PNG), and 4957 (53%) of them were followed up until delivery (Fig 1). Table 2 the baseline characteristics of the study participants by site (S1 Table). Mean age of study women was 23.6 years [standard deviation (SD) = 5.6], mean gestational age 23 weeks (SD = 8); nearly 40% (3759/9468) of the women were primigravidae. At enrolment, overall mean Hb level was 10.5 g/dL (SD 1.8), and 57% (5201/9105) of women were anemic (Hb<11g/dL). Around 70% of women (6471) were enrolled at their first ANC visit versus at later ANC visit.

**Table 2:** Baseline characteristics of study women at enrolment.

		Overall		Colombia		Guatemala		Brazil		India		Papua New Guinea	
**Participants** (N)		9388		2043		2009		1657		1982		1697	
**Age** (years) *		23.6 (5.6) [9345]		22.2 (5.7) [2043]		24.4 (6.6) [2001]		23.5 (6.2) [1655]		23.1 (3.4) [1980]		25.0 (5.4) [1677]	
**Gestational age** (weeks) *		24 (8) [8539]		21 (9) [2042]		26 (8) [1978]		22 (8) [1624]		24 (6) [1979]		23 (5) [915]	
		**n**	**(%)**	**n**	**(%)**	**n**	**(%)**	**n**	**(%)**	**n**	**(%)**	**n**	**(%)**
**Gestational age** †	1st trimester	891	(11)	453	(22)	131	(7)	231	(14)	35	(2)	41	(4.4)
	2nd trimester	3608	(42)	805	(39)	623	(31)	731	(45)	984	(50)	465	(51)
	3rd trimester	4040	(47)	785	(38)	1224	(62)	662	(41)	960	(49)	409	(45)
**Weight** (kg) *		57.7 (10.2) [9224]		58.1 (10.6) [2015]		58.6 (9) [1999]		61.8 (11.8) [1608]		54.6 (9.5) [1942]		55.7 (8) [1660]	
**Height** (cm) *		154.1 (6.1) [9328]		156.3 (5.8) [2025]		150.9 (5.7) [2005]		154.1 (5.9) [1654]		155 (5.9) [1980]		154.3 (6) [1664]	
**Haemoglobin** (gr/dL)		10.5 (1.8) [9105]		11.2 (1.7) [2033]		11.2 (1.3) [1761]		11.5 (1.1) [1642]		9.1 (1.6) [2025]		9.5 (1.5) [1644]	
		**n**	**(%)**	**n**	**(%)**	**n**	**(%)**	**n**	**(%)**	**n**	**(%)**	**n**	**(%)**
**Gravidity**	Primigravidae	3719	(40)	672	(33)	726	(36)	532	(32)	1001	(51)	788	(47)
	1 to 3 pregnancies	3680	(39)	863	(42)	681	(34)	663	(40)	893	(45)	580	(34)
	4 or > pregnancies	1972	(21)	508	(25)	596	(30)	462	(28)	87	(4)	319	(19)
**Overall anaemia at baseline** (Hb<11 g/dl)		5201	(57.1)	867	(43)	661	(37.5)	496	(30)	1790	(88.3)	1387	(84.3)
**Severe anaemia at baseline** (Hb<7 g/dL)		288	(3.1)	9	(0.4)	14	(0.8)	4	(0.2)	175	(8.6)	86	(5.2)
**History of fever last 24h**		187	(2.2)	33	(2)	32	(2)	42	(3)	11	(1)	69	(7.5)
**Fever** (axillary temperature ≥ 37.5°C)		97	(1)	16	(1)	34	(2)	5	(0.4)	6	(0.3)	36	(2.2)
**Previous malaria episodes during current pregnancy**		292	(3.4)	85	(4)	3	(0.1)	22	(1.3)	0	(0)	182	(19.8)
**Slept under a bednet the night before**		3428	(40)	778	(38)	1824	(91)	119	(7)	1	(0.05)	706	(77)
**Indoor residual spraying**		2178	(29.4)	861	(42)	650	(32)	593	(36)	0	(0)	74 ‡	(4)
**Antimalarials taken during pregnancy** (as treatment)		249	(2.9)	80	(4)	4	(0.4)	22	(1.3)	0	(0)	143	(16.7)
**Taking malaria prophylaxis**		45	(0.5)	11	(0.5)	1	(0.05)	2	(0.1)	0	(0)	31	(2)
		**n/N**	**(%)**	**n/N**	**(%)**	**n/N**	**(%)**	**n/N**	**(%)**	**n/N**	**(%)**	**n/N**	**(%)**
**Positive by microscopy**	*P*. *vivax*	73/9299	(0.8)	12/2025	(0.6)	2/2005	(0.1)	5/1584	(0.3)	25/2020	(1.2)	29/1665	(1.7)
	*P*. *falciparum*	124/9305	(1.5)	12/2027	(0.6)	0/2005	(0)	0/1587	(0)	1/2021	(0.05)	111/1665	(6.7)
	Positive for any species	197/9326	(2.1)	24/2029	(1.2)	2/2005	(0.1)	5/1587	(0.3)	26/2021	(1.3)	140/1684	(8.3)
**Positive by PCR**	*P*. *vivax*	124/1455	(8.5)	18/300	(6)	42/300	(14)	25/299	(8.4)	3/298	(1)	36/258	(13.9)
	*P*. *falciparum*	80/1157	(6.9)	9/300	(3)	35/300	(11.6)	6/299	(2)	NA	(.)	30/258	(11.6)
	Positive for any species	182/1165	(15.6)	26/300	(8.7)	63/300	(21)	30/299	(10)	NA	(.)	63/266	(23.7)

* Arithmetic Mean (SD) [n].

^†^ 1st trimester: 0–12 weeks, 2nd trimester: 13–24 weeks, 3rd trimester: 25–40 weeks.

^‡^ No indoor residual spraying (IRS) campaigns in the study area.

### Prevalence of *P*. *vivax* mono-infection

**Fig 2:**
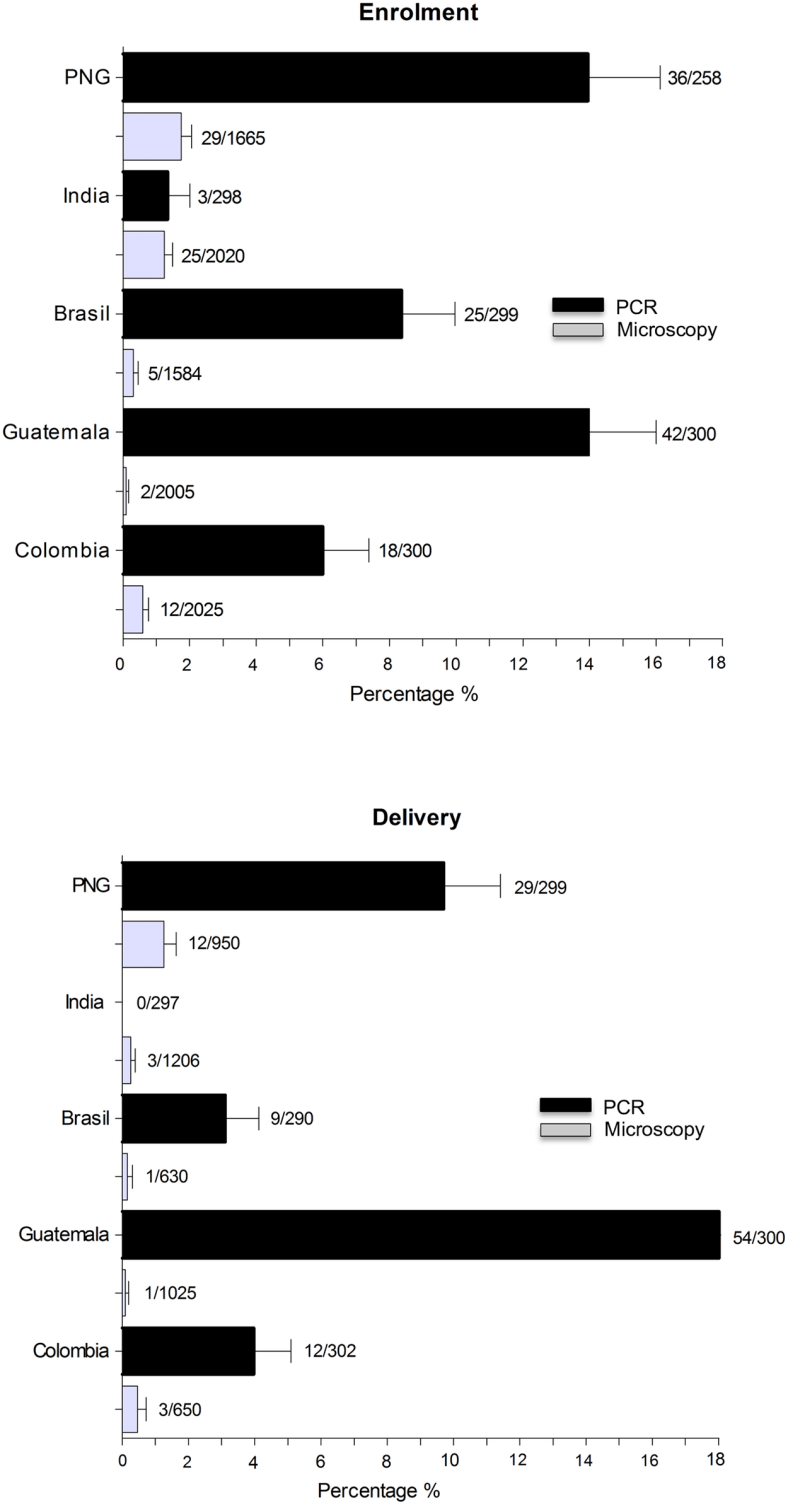
Prevalence of *P*. *vivax* infection.

The overall prevalence of *P*. *vivax* mono-infection detected by microscopy was 0.8% (73/9299) at enrolment and 0.4% (20/4461) at delivery. Of those women who were parasitemic for *P*. *vivax* 57.6% (42/73) were symptomatic at enrolment and 20% (4/20) at delivery. The prevalence of *P*. *vivax* mono-infection by PCR assessed in a subsample of women was 8.5% (124/1455) at enrolment and 6.9% (104/1488) at delivery, and of those 9.7% (12/124) and 4.8% (5/104) reported symptoms, respectively (Fig 2).

### Prevalence of *P*. *falciparum* mono-infection

**Fig 3:**
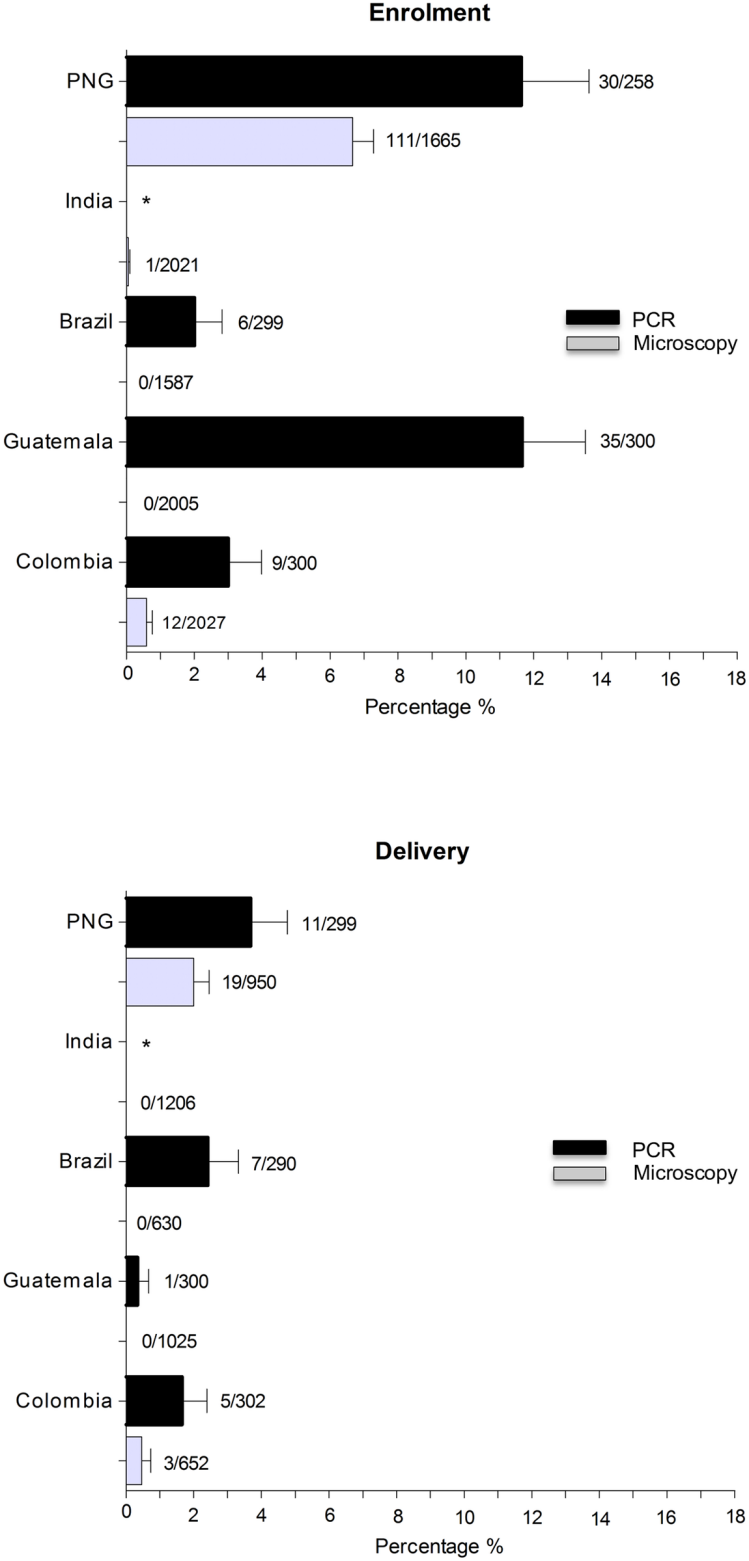
Prevalence of *P*. *falciparum* infection. * Not available data. Technical problems in the performance of the molecular analyses (PCR) for *P*. *falciparum* occurred at the reference laboratory in India. No biological specimens remained available after that sufficient to repeat the analyses in an alternative laboratory.

The overall prevalence of *P*. *falciparum* mono-infection detected by microscopy was 1.3% (124/9305) at enrolment and 0.5% (22/4463) at delivery. Of those women who were parasitemic for *P*. *falciparum* 16.1% (20/124) were symptomatic at enrolment and 27.3% (6/22) at delivery. No *P*. *falciparum* infection was detected by microscopy in GT and BR neither at enrolment or delivery, nor in India at delivery. The prevalence of *P*. *falciparum* mono-infection by PCR assessed in a subsample of women was 6.9% (80/1157) at enrolment and 2% (24/1191) at delivery, and of those 10% (8/80) and 12.5% (3/24) reported symptoms, respectively (Fig 3).

### Mixed infections

There were no mixed infections (*P*. *vivax* plus *P*. *falciparum*) detected by microscopy during pregnancy or at delivery in CO, GT, BR and IN. In PNG mixed infections were observed in 9/818 (1.1%) of placental impression smears only. The prevalence of mixed infections detected by PCR was 2.2% (26/1165), 0.3% (4/1191), 0.3% (1/379) at enrolment, delivery, and in cord blood, respectively. No mixed infections were detected in the placental blood.

Data on malaria infection in the placenta are shown in Table 3. Data on congenital malaria can be found in Table 4.

**Table 3:** Malaria infection in the placenta.

	Overall		Colombia		Guatemala		Brazil		India		Papua New Guinea	
**Positive by microscopy** (impression smear)	**n/N**	**(%)**	**n/N**	**(%)**	**n/N**	**(%)**	**n/N**	**(%)**	**n/N**	**(%)**	**n/N**	**(%)**
*P*. *vivax*	13/3695	(0.3)	0/631	0	0/637	(0)	1/709	(0.14)	0/900	(0)	12/818	(1.5)
*P*. *falciparum*	32/3695	(0.8)	3/631	(0.5)	0/637	(0)	0/709	(0)	0/900	(0)	29/818	(3.5)
Positive for any species*	36/3702	(0.9)	3/635	(0.5)	0/637	(0)	1/709	(0.14)	0/903	(0)	32/818	(3.9)
**Positive by PCR**												
*P*. *vivax*	19/503	(3.7)	2/100	(2)	14/102	(13.7)	0/95	(0)	0/100	(0)	3/106	(2.8)
*P*. *falciparum*	2/403	(0.5)	1/100	(1)	0/102	(0)	0/95	(0)	−		1/101	(0.9)
Positive for any species *	21/403	(5.2)	3/100	(3)	14/102	(13.7)	0/95	(0)	−		4/106	(3.7)
**Classification of placental infection by histology** †												
Active (acute + chronic)	49/1144	(4.3)	1/119	(0.8)	0/87	(0)	0/85	(0)	3/139	(2.2)	45/714	(6.3)
Past	92/1144	(8)	16/119	(13.4)	1/87	(1.1)	1/85	(1.2)	1/139	(0.7)	73/714	(10.2)
Not infected	1003/1144	(87.6)	102/119	(85.7)	86/87	(98.8)	84/85	(98.8)	135/139	(97.1)	596/714	(83.5)
**Placental inflammation**												
Intervillous inflammation	158/1150	(13.7)	7/121	(5.7)	19/88	(21.6)	12/85	(14.1)	6/139	(4.3)	114/717	(15.9)
Infarcted areas	9/1150	(0.8)	7/121	(5.7)	1/88	(1.1)	0/85	(0)	1/139	(0.7)	0/717	(0)

* Whether P. vivax and/or P. falciparum. There were a total of 9 mixed infections (Pv+Pf) on the placental impression smear (by microscopy).

^†^ Acute: parasites with no/mild pigment. Chronic: parasites + significant pigment deposition. Past: pigment with absence of parasites. Non-infected: no evidence of parasites or pigment.

**Table 4:** Congenital malaria cases per country.

	Overall		Colombia		Guatemala		Brazil		India		Papua New Guinea	
***Cord blood***												
Positive by microscopy	**n/N**	**(%)**	**n/N**	**(%)**	**n/N**	**(%)**	**n/N**	**(%)**	**n/N**	**(%)**	**n/N**	**(%)**
*P*. *vivax* infection	2/4040	(0.05)	0/618	(0)	1/900	(0.1)	0/643	(0)	0/1183	(0)	1/696	(0.1)
*P*. *falciparum* infection	9/4040	(0.22)	1/618	(0.16)	0/900	(0)	0/643	(0)	0/1183	(0)	8/696	(1.15)
Any *Plasmodium* species	11/4061	(0.27)	1/636	(0.15)	1/900	(0.1)	0/643	(0)	0/1183	(0)	9/699	(1.3)
Positive by PCR												
*P*. *vivax* infection	13/497	(2.6)	0/100	(0)	13/101	(12.8)	0/100	(0)	0/100	(0)	0/96	(0)
*P*. *falciparum* infection	7/385	(1.8)	3/100	(3)	2/101	(1.9)	1/100	(1)	−		1/84	(1.2)
Any *Plasmodium* species	20/385	(5.2)	3/100	(3)	15/101	(14.8)	1/100	(1)	−		1/96	(1.04)
***Newborn blood***												
Positive by microscopy												
*P*. *vivax* infection	1/4302	(0.02)	0/600	(0)	0/975	(0)	0/628	(0)	1/1174	(0.08)	0/925	(0)
*P*. *falciparum* infection	2/4303	(0.05)	0/600	(0)	0/975	(0)	0/629	(0)	0/1174	(0)	2/925	(0.2)
Any *Plasmodium* species	3/4309	(0.07)	0/600	(0)	0/975	(0)	0/629	(0)	1/1174	(0.08)	2/931	(0.2)

### Incidence of malaria

Data on incidence of malaria is shown in Table 5. The incidence rate of *P*. *vivax* infection detected by microscopy was not associated with parity (Incidence Rate Ratio-IRR for multigravidae-women with 4 or more pregnancies-, 1.14 [95% CI 0.70–1.87]; p = 0.619). For *P*. *falciparum* the IRR for multigravidae was 0.39, [95% CI 0.25–0.62]; p<0.001). A total of 898 passive case detection visits (528 in PNG, 183 in CO, 75 in BR, 62 in GT and 50 in IN) were registered among study women who reported symptoms or had signs suggestive of malaria infection. Of those, 823 women had a blood smear collected, and in 141 (17%) of them malaria infection was confirmed by microscopy; 68 cases were due to *P*. *vivax*, 72 cases were due to *P*. *falciparum*, and one case was due to *P*. *malariae*. In 85% (117/137) malaria episodes women were found to be anemic (Hb<11g/dL) and 14% (19/137) severely anemic (Hb<7g/dL). Only 8 women with clinical malaria (2 with *P*. *vivax* and 6 with *P*. *falciparum* infection, and all from CO) required hospital admission (data from admitted women in PNG not available). On average, 2% (86/4227) of study women reported at delivery a history of having had a malaria episode during the current pregnancy, and 2.3% (90/3830) having taken antimalarial drugs during the same pregnancy. See S2 Table for incidence data disaggregated by PCD or ADI, overall and by country.

**Table 5:** Incidence of malaria overall and by species and country.

	Overall			Colombia			Guatemala			Brazil			India			Papua New Guinea		
	Events	PYAR*	Rate	Events	PYAR	Rate	Events	PYAR	Rate	Events	PYAR	Rate	Events	PYAR	Rate	Events	PYAR	Rate
**Detected through PCD or ADI** †																		
Incidence of *P*. *vivax* malaria	121	1679	0.072	27	366	0.073	3	324	0.009	14	313	0.044	23	381	0.060	54	295	0.183
Incidence of *P*. *falciparum* malaria	187	1668	0.112	14	369	0.037	0	324	0.000	0	315	0.000	1	382	0.002	172	278	0.618
Incidence of any *Plasmodium* malaria	306	1655	0.184	41	364	0.112	3	324	0.009	14	313	0.044	24	381	0.062	224	273	0.821

* PYAR: person-years at risk.

^†^PCD: Passive Case Detection at the outpatient clinic. ADI: Active Detection of Infection at the ANC scheduled visits.

### Plasmodium parasite densities

Overall, parasite densities in pregnant women throughout the study were low in most cases. The geometric mean density (GMD) for *P*. *vivax* infections on maternal peripheral blood at any time during the study was 1275 parasites/μL (95% CI 906.5–1643.5); being 385 parasites/μL (95% CI 226.9–543.1) and 2396 parasites/μL (95% CI 1579.5–3212.5) the GMDs for *P*. *vivax* asymptomatic and symptomatic infections, respectively. The GMD for *P*. *falciparum* infections on maternal peripheral blood was 1678 parasites/μL (95% CI 1268.6–2087.4); being 918 parasites/μL (95% CI 681.7–1154.3) and 4087 parasites/μL (95% CI 2362.1–5811.8) the GMDs for *P*. *falciparum* asymptomatic and symptomatic infections, respectively.

Parasite densities on placental blood ranged for *P*. *vivax* from 0.6 parasites/μL in the positive case detected in Brazil to 25.5 parasites/μL arithmetic mean (SD 31.8) in PNG. For *P*. *falciparum* densities in the placenta ranged from 21.4 parasites/μL arithmetic mean (SD 25.5) in CO up to 6255 parasites/μL arithmetic mean (SD 7845) in placentas from PNG.

### Pregnancy outcomes

Overall, 10.2% (505/4943) of babies born to women enrolled in the study had LBW (range 2.8% in CO– 16.5% in PNG), and the prevalence of pre-term birth was 12.6% (455/3590) across countries (Table 6). At delivery, on average 53.4% (2285/4278) of study women were anemic (range 17.8% in BR– 88.3% in IN), and 4.5% of them (193/4278) were severely anemic. Most of the documented deliveries (96%) took place at a health facility (hospital/health center) attended by skilled staff. During the study follow up there were three maternal deaths and their clinical diagnoses at the time of death were recorded; one in CO due to *P*. *falciparum* malaria, one in PNG due to post-partum hemorrhage with no evidence of malaria at any time during pregnancy, and one in IN associated with *P*. *vivax* malaria and anemia.

**Table 6:** Pregnancy outcomes.

	Overall		Colombia		Guatemala		Brazil		India		Papua New Guinea	
**Number of deliveries registered** (N)	4957		779		1025		860		1294		999	
Gestational age (weeks) *	38.6 (3.0) [3590]		39.3 (2.3) [276]		39.5 (2.2) [948]		39.4 (1.5) [731]		38.5 (2.5) [576]		38.1 (4.3) [1059]	
Birth weight *	3042 (507) [4943]		3235 (406) [776]		3127 (486) [992]		3249 (517) [869]		2812 (443) [1259]		2921 (511) [1047]	
Haemoglobin mother (g/dL) *	10.4 (2.9) [4218]		10.8 (1.7) [669]		11.4 (1.6) [929]		12.0 (1.5) [696]		8.9 (1.6) [886]		9.6 (4.5) [1038]	
	**n/N**	**(%)**	**n/N**	**(%)**	**n/N**	**(%)**	**n/N**	**(%)**	**n/N**	**(%)**	**n/N**	**(%)**
Pre-term birth (<37 weeks)	455/3590	(12.6)	33/276	(12)	42/948	(4.4)	28/731	(3.8)	132/576	(23)	220/1059	(20.7)
Low birth weight (<2500g)	503/4901	(10.2)	22/776	(2.8)	68/992	(6.8)	64/836	(7.6)	176/1250	(14)	173/1047	(16.5)
***Mother***												
Fever (axillary temperature ≥ 37.5°C)	142/4699	(3)	15/777	(1.9)	25/1025	(2.4)	11/719	(1.5)	41/1191	(3.4)	50/987	(5.06)
History of fever last 24h	38/4249	(0.9)	5/774	(0.6)	10/1024	(0.9)	2/726	(0.3)	0/1185	(0)	21/540	(3.9)
Overall anaemia at delivery (Hb<11 g/dl)	2285/4278	(53.4)	345/669	(51.5)	337/1016	(33.1)	119/679	(17.8)	773/876	(88.3)	711/1038	(68.5)
Severe anaemia at delivery (Hb<7 g/dL)	193/4278	(4.5)	7/669	(1.04)	7/1016	(0.7)	5/679	(0.72)	66/876	(7.5)	108/1038	(10.4)
***Newborn***												
Prevalence of congenital abnormalities	27/4890	(0.55)	8/737	(1.1)	6/1011	(0.6)	3/851	(0.3)	2/1181	(0.2)	9/1110	(0.1)
Fever (axillary temperature ≥ 37.5°C)	83/4644	(1.8)	2/735	(0.3)	7/979	(0.7)	0/707	(0)	64/1179	(5.4)	10/1044	(0.9)

* Arithmetic Mean (SD) [n]. Assessment of gestational age in GT, BR and PNG was done by Ballard method, an in Colombia and India by ultrasound.

### Impact of *P*. *vivax* and *P*. *falciparum* malaria on maternal and newborn outcomes

At enrolment women with *P*. *vivax* or *P*. *falciparum* clinical malaria were more than 5-times as likely to be anemic compared to non-infected women (adjusted OR, 5.48, [95% CI 1.82–16.41]; p = 0.009), and adjusted OR, 5.57, [95% CI 1.22–25.34]; p = 0.012, respectively) (Table 7). In contrast, those women with clinical malaria at delivery, whether infected with *P*. *vivax* (p = 0.754) or *P*. *falciparum* (p = 0.122), did not show an increased risk of anemia at the same time point compared to non-infected or infected-asymptomatic women. No association was found between *P*. *vivax* or *P*. *falciparum* clinical malaria at enrolment (p = 0.100, and p = 0.760, respectively) or at delivery (p = 0.120, and p = 0.060, respectively) and an increased risk of LBW.

**Table 7:** Impact of *P*. *vivax* and *P*. *falciparum* malaria on maternal anemia and low birth weight.

	*P*. *vivax*						*P*. *falciparum*					
	Maternal anaemia *			Low birth weight			Maternal anaemia			Low birth weight		
	Adjusted OR †	(95% CI)	p-value	Adjusted OR	(95% CI)	p-value	Adjusted OR	(95% CI)	p-value	Adjusted OR	(95% CI)	p-value
**At enrolment**												
Non-infected	1		**0.009**	1		0.350	1		**0.001**	1		0.092
Asymptomatic malaria infection	0.96	(0.37–2.44)		0.92	(0.21–4.14)		4.01	(1.59–10.11)		2.03	(1.07–3.85)	
Clinical malaria	5.48	(1.83–16.41)		2.29	(0.74–7.04)		5.57	(1.22–25.34)		1.39	(0.16–12.03)	
**At delivery**												
Non-infected	1		0.754	1		0.298	1		0.122	1		**0.004**
Asymptomatic malaria infection	0.66	(0.22–1.96)		1.01	(0.22–4.63)		8.19	(1.08–62.38)		4.52	(1.63–12.49)	
Clinical malaria	1.05	(0.07–15.58)		9.39	(0.56–158.25)		1.29	(0.23–7.18)		4.29	(0.70-26-24)	
Non-infected	1		0.384	1		0.895	1		**<0.001**	1		**0.001**
Microscopic infection	0.62	(0.22–1.81)		0.90	(0.20–4.10)		4.07	(1.93–8.58)		4.28	(1.75–10.44)	
Non-infected	1		0.717	1		0.110	1		0.402	1		0.359
Sub-microscopic infection ‡	1.16	(0.52–2.59)		0.52	(0.23–1.16)		2.93	(0.24–36.23.16)		1.97	(0.46–8.46)	

* Maternal anaemia: Hb< 11 g/dL.

^†^ Adjusted Odds Ratio (OR) for site and previous malaria episodes.

^‡^ Nested case-control study.

Women with *P*. *vivax* malaria at delivery detected by microscopy did not have an increased risk of anemia (adjusted OR, 0.62, [95% CI 0.22–1.81]; p = 0.384) or LBW (adjusted OR, 0.90, [95% CI 0.20–4.10]; p = 0.895), compared to uninfected women. However, those women with *P*. *falciparum* malaria showed an increased risk for both anemia (adjusted OR, 4.07, [95% CI 1.93–8.58]; p = 0.0002) and LBW (adjusted OR, 4.28, [95% CI 1.75–10.44]; p = 0.0014). In the nested case-control sub-analysis, women with *P*. *vivax* submicroscopic infection at delivery did not show an increased risk of moderate-severe anemia (adjusted OR, 1.16, [95% CI 0.52–2.59]; p = 0.717) or LBW (adjusted OR, 0.52, [95% CI 0.23–1.16]; p = 0.110) compared to uninfected women (negative by microscopy and by PCR). Likewise, *P*. *falciparum* submicroscopic infections at delivery were not associated with moderate-severe anemia (adjusted OR, 2.93, [95% CI 0.24–36.23]; p = 0.402) or LBW (adjusted OR, 1.97, [95% CI 0.46–8.46]; p = 0.359).

Malaria infection in the placenta (whether detected by impression smear or PCR) was not associated with an increased risk of LBW (adjusted OR, 0.67, [95% CI 0.07–6.18]; p = 0.725 for *P*. *vivax*, and adjusted OR, 8.61, [95% CI 0.79–94.09]; p = 0.077, for *P*. *falciparum*) or anemia (adjusted OR, 2.04, [95% CI 0.70–5.92]; p = 0.189 for *P*. *vivax*, and adjusted OR, 0.77, [95% CI 0.16–3.67]; p = 0.746, for *P*. *falciparum*). In contrast, a statistically significant association was found for placental infections detected by histology with LBW (adjusted OR, 2.36, [95% CI 1.48–3.76]; p = <0.001) and anemia (adjusted OR, 1.66, [95% CI 1.09–2.51]; p = 0.017). Histology did not allow distinguishing between species, but of the infected placentas according to histology a higher proportion were positive for *P*. *falciparum* (11.4%) compared to *P*. *vivax* (4.8%) by impression smear or PCR.

## Discussion

This is, to our knowledge, the first multicenter prospective observational study on the burden of *P*. *vivax* in pregnant women from different malaria endemic areas. Overall, the proportion of pregnant women attending the ANC who were infected with vivax malaria detected by microscopy was low across the different endemic settings included in the study. However, *P*. *vivax* infection in pregnancy was associated with negative consequences for maternal health in those that were symptomatic, which may have a negative impact also for the health of the neonate. Importantly, most of the *P*. *vivax* infections in pregnancy were undetected with microscopy, the routinely used screening method across study sites.

The majority of the information on *P*. *vivax* in pregnancy comes from studies carried out in Asia. [19] There is much less information from Latin America, where *P*. *vivax* is responsible for 65 to 100% of malaria cases;[26] a community-based study conducted in the Brazilian Amazon in 2002 showed that 1.9% of the pregnant women were infected with *P*. *vivax*,[27] and a study from Colombia conducted between 2005 and 2011 reported that 11.6% of pregnant women were infected at delivery with *P*. *vivax*. [28] However, in the current study, the frequency of *P*. *vivax* infection detected by microscopy was below 2% in all countries and at all time points. Recent efforts in the deployment of malaria preventive measures, as it was the case of Guatemala in this study, together with the introduction of new more effective antimalarial drugs for case management, may have played a role in the decrease in the prevalence of infections observed in this study below microcopy-detection levels, but still allowing a considerable proportion of the population harboring sub-microscopic parasitemia levels.

Mixed infections were rare, except in PNG, which is different from the situation in Africa where most non-falciparum infections in pregnancy are mixed infections. [29] Histology was the method showing the highest sensitivity for detection of malaria infection in the placenta compared to microscopy and PCR, and the presence of malaria pigment more frequent than of parasites in the infected placentas.

In most study countries, optical microscopy is used for case detection of malaria infection,[26] and for routine screening of malaria at ANC visits (where the latter is recommended). However, the fact that PCR methods detected significantly more infections than microscopy suggest that many infections were undetected and that microscopy is not a sensitive method for routine screening of malaria, particularly in *P*. *vivax* endemic areas due to the generally low parasite densities. [30]

The available data on the impact of *P*. *vivax* in pregnancy are scarce, and they have rarely been analysed disaggregated from the effects of *P*. *falciparum* infection. Thus, understanding the main effects of *P vivax* monoinfection in pregnancy was a main objective of this study. It was observed that during pregnancy pregnant women with clinical *P*. *vivax* monoinfection had a 5-fold higher risk of having anemia (Hb<11g/dL) compared to non-infected women. In malaria endemic areas, there is evidence on the impact of maternal anemia on maternal mortality and morbidity,[31] as well as on neonatal and infant health and survival. [6, 8] In contrast, asymptomatic *P*. *vivax* infection was not associated with an increased risk of maternal anemia of any degree. The evidence from this study also indicated that it is rare for *P*. *vivax*, and for *P*. *falciparum* to establish an infection in the newborn.

The two maternal deaths reported in Colombia and India were associated with *P*. *falciparum* and *P*. *vivax* infections, respectively, underpinning that malaria in pregnancy can be a contributor to maternal mortality in these low transmission settings. These results claim the maternal and neonatal health programs in *P*. *vivax* endemic areas to recognize that vivax malaria is associated with negative consequences for the health of pregnant women and consequently for that of the neonate. Clinical and epidemiologic surveillance systems need to be integrated into maternal and reproductive health programs to detect malaria cases in pregnant women for prompt and effective case management.

The results of this study can also contribute to evaluate whether current strategies for malaria control during pregnancy adopted in areas where *P*. *vivax* is predominant are adequate, and if they are aligned with most recent evidence. In this study, performance of passive surveillance was better in detecting *Plasmodium* infections (17% positivity by blood smear among women with malaria symptoms) compared to active detection at routine visits (2.1% and 0.94% of women infected at enrolment and at delivery visits, respectively). As malaria transmission diminishes, the approach to the detection of infected pregnant women may change from active detection at each ANC visit to passive detection to capture the infections (febrile, symptomatic cases) that are mostly associated with poor pregnancy outcomes. As malaria burden decreases the sustainability of an active approach might be questionable, as shown in some countries in the Americas, nor cost-effective. Surveillance is of particular relevance for those countries that are in the pathway towards malaria elimination and will need to shift from measuring reductions in morbidity and mortality, to detecting infections and measuring transmission.

*P*. *vivax* is becoming the dominant species in low transmission areas. [26] Accurate data on *P*. *vivax* burden depending on the level of malaria endemicity is crucial to designing effective malaria control and elimination policies. However, *P*. *vivax* poses unique epidemiological challenges. In contrast to *P*. *falciparum*, *P*. *vivax* can arise from dormant hypnozoites leading to relapses weeks or months after, that can be indistinguishable from a primary infection. [32] The prevention of these relapses is considered to be the most important strategy for interrupting *P*. *vivax* transmission. [33] In pregnant women the use of primaquine, a member of the 8-aminoquinoline family and the only drug currently licensed for radical cure of *P*. *vivax* malaria, is contraindicated for safety reasons, putting them at a higher risk of clinical relapses. The role that pregnant women may play as asymptomatic parasite reservoirs needs to be further investigated, as this might represent a limitation for effective malaria control and elimination.

There are limitations to this study. The study was able to capture data and collect samples from pregnant women at the time of delivery for more than half of study women, but this still represented a low follow-up rate. This limitation is not uncommon in health facility based studies from settings where most women deliver at home,[34] as it was the case for most of study sites. Comparison of baseline characteristics between complete and lost to follow-up women showed that differences were related primarily to characteristics of the site or site-related, Although statistical analyses were adjusted by site, these dissimilarities need to be carefully considered as this may imply that the findings might not be generalized to the women who do not deliver at the health facility.

The evaluation of gestational age ultrasound assessment was not available at those facilities, and the Ballard′s method was used in some of the study countries. This might represent a limitation, as the latter is not as reliable method as ultrasound is. PCR assays for detection of malaria infection were performed at a different laboratory in India due to regulatory issues that did not allow analyzing samples at the study central laboratory at the ISS in Rome. Although the same standardized protocols were used in both laboratories, and that internal and external validations were performed in a subset of samples, this might have introduced some potential bias by pooling of data obtained from different laboratories. In addition, much of the data shown in this study is reported as aggregate to provide an estimate of the wider geographic situation. However, the interpretation of these data has to take into consideration the differences in some of the estimates across sites. Also, some women (2.4%) reported having received antimalarial treatment during pregnancy. Though analyses of the impact of microscopic and sub-microscopic infection were adjusted by previous malaria episodes and study site, it needs to be taken into consideration as this may have impacted the results. Lastly, the low proportion of microscopically detected infections and that PCR determination of malaria infection could not be performed in all samples did represent a limitation for the pooled analysis on the impact of microscopic and submicroscopic infections. The lack of association of submicroscopic and placental infections (except those detected by histology) with anemia and LBW needs to be viewed with caution as it could be related to a sample size issue, and thus not being enough placental infections to detect a clinically important difference.

### Conclusions

This multicenter study in representative areas endemic for vivax malaria shows that the prevalence of *P*. *vivax* malaria was overall low across study sites. However, the prevalence of submicroscopic infections was significant in some areas, which may have implications for detection of malaria infections with the currently used diagnostic tools. Clinical episodes due to *P*. *vivax* infection were associated with maternal anemia, which may be also detrimental for infant’s health. These findings can be relevant for guiding maternal health programs in settings where *vivax* malaria is endemic, as well as for monitoring, evaluation and surveillance activities in countries that are in the pathway towards malaria elimination.

## Supporting information

S1 File Further details of the methods used in the histological evaluation of placentas. (PDF)

S1 Table Comparison of baseline characteristics between lost to follow-up women and women with complete follow-up. (PDF)

S2 Table Incidence of malaria detected through passive case detection (PCD) and through active detection of infection (ADI), overall and by species and country. (PDF)

S1 Checklist STROBE checklist. (PDF)
