# Supplementary material for: Burden and impact of Plasmodium vivax in pregnancy: A multi-centre prospective observational study
Source: PLoS Negl Trop Dis. 2017 Jun 12;11(6):e0005606. doi: 10.1371/journal.pntd.0005606 (PMC5481034; doi:10.1371/journal.pntd.0005606)
Supplement: S1 File — (PDF) [file pntd.0005606.s001.pdf]

**S1 File. Further details of the methods used in the histological evaluation of the placentas**

In a randomly selected subsample of women (10%) a placental biopsy was collected from the maternal side of the placenta, kept at 4ºC in 10% neutral buffer formalin, processed for histological examination, and stained with haematoxylin and eosin as previously described[1]

1.1. Procedures (extracted from the SOP for the evaluation of histological sections to detect placental malaria) [2,3]

1.1.1. Introduction

- All samples for histological evaluation were received from the laboratory as:
  - Hematoxylin and eosin stained slide
  - Paraffin block
- All slides and blocks were adequately identified with the women study number
- In every case a histological evaluation form was filled

1.1.2. Filling the sample identification and evaluation data (evaluation form, part I)

- The study identification number (item 1)
- The evaluator code and the date of evaluation (item 2). If an agreement evaluation was done by two observers the number of each of them were recorded
- If a quality control observation was done and "x" was written (item 3)
- The controller code and the date of evaluation (item 4).

1.1.3. Evaluation of the preservation, adequacy of the sample and the technique used in the evaluation (evaluation form, part II)

- Was the evaluation conducted under non-polarizing light? (1=yes; 2=no) (item 5)
- Was the evaluation conducted under polarizing light? (1=yes; 2=no) (item 6)
- Was the evaluation done on Hematoxylin and eosin stain? (1=yes; 2=no) (item 7)
- Was the evaluation done on Giemsa' stain? (1=yes; 2=no) (item 8)
- Evaluate the presence or absence of autolysis (characterized by poor preservation of the tissue, with absence of nuclei): 1=absent, 2= mild, 3=moderate, 4=severe (item 9)
- Evaluate the presence or absence of formalin pigment (when present pigment is detected in a widespread location): 1=absent, 2= mild, 3=moderate, 4=abundant (item 10)
- Evaluate the amount of maternal erythrocytes intervillous space: 1=absent, 2= scant, 3=moderate, 4=abundant (item 11)
- Evaluate the presence or absence of decidua basalis in the sample: 1=absent; 2=present (item 12)

- Evaluate the presence or absence of amnios in the sample: 1=absent; 2=present (item 13)

1.1.4. Evaluation of the presence or absence of malarial parasites and hemozoin (evaluation form, part III)

- The whole slide at low magnification was initially assessed [40x, 100x (objectives of 4x or 10x)] (using if possible polarized and non-polarized light) looking for parasites and pigment
- Careful evaluation at 1000x magnification (objective 100x with immersion oil) looking for parasitized erythrocytes
- Are there 500 maternal erythrocytes in the slide? (1=yes; 2=no) (item 14)
- If less than 500 maternal erythrocytes are identified, how many? (write the number) (item 15)
- Are there parasitized maternal erythrocytes in the intervillous space (1=yes; 2=no) (item 16)
- If parasites were identified, look for the area with more parasites, count 500 erythrocytes and determine the percentage of parasitized maternal erythrocytes. Then, the percentage was counted and written (percentage=number of parasitized maternal erythrocytes identified*100/500; if less than 500 maternal erythrocytes are identified, percentage=number of parasitized maternal erythrocytes identified*100/number of maternal erythrocytes present) (item 17)
- The slide was scanned at low magnification [40x, 100x (objectives of 4x or 10x)] using (if possible) polarized and non-polarized light). If no polarized light was available and no pigment was identified, the scan was repeated at 400 magnification (objective 40x)
- Record whether malarial pigment was identified or not. If malarial pigment was identified, the amount of pigment present was semiquantitatively evaluated in the placenta (item 18)
    - 1= no pigment identified
    - 2 (mild): few spots identified focally at high magnification [400 x (objective 40x)]
    - 3 (Moderate): coarse deposits identified focally at high magnification [100x (objective 10x)], but focally located
    - 4 (Abundant): large and coarse spots identified at low magnification [100x (objective 10x)], diffusely distributed
- Are there free macrophages in the maternal space with malarial pigment (1=yes; 2=no) (item 19)
- Is there hemozoin deposition in fibrin (either in macrophages or free) (1=yes; 2=no) (item 20)
- Record whether parasites or malarial pigment are present in fetal erythrocytes or villi (1=yes; 2=no) (item 21)

1.1.5. Evaluation of other abnormalities (evaluation form, part IV)

- Evaluation of the intervillous inflammation: Look at low magnification for the area with more white cells. Use high magnification (400, objective 40x) to count the number of white cells per area (item 22)
    - 1=<5

- 2=5-10
- 3=10-25
- 4=>25

- Evaluate the presence of infarcted areas (item 23)
  - 1= absent
  - 2= present
- Evaluate the presence of chorioamnionitis (item 24)
  - 1= absent
  - 2= present
  - 3=not applicable (no amnios present in the slide)

1.1.6. References


1. Ismail MR, Ordi J, Menendez C, Ventura PJ, Aponte JJ, Kahigwa E, Hirt R, Cardesa A, Alonso PL. Placental pathology in malaria: a histological, immunohistochemical, and quantitative study. Hum Pathol. 2000 Jan;31(1):85-93.
2. Bulmer JN, Rasheed FN, Francis N, Morrison L, Greenwood BM. Placental malaria. I. Pathological classification. Histopathology. 1993 Mar;22(3):211-8.
3. Bulmer JN, Rasheed FN, Morrison L, Francis N, Greenwood BM. Placental malaria. II. A semi-quantitative investigation of the pathological features. Histopathology. 1993 Mar;22(3):219-25.
