# Supplementary material for: Burden and impact of Plasmodium vivax in pregnancy: A multi-centre prospective observational study
Source: PLoS Negl Trop Dis. 2017 Jun 12;11(6):e0005606. doi: 10.1371/journal.pntd.0005606 (PMC5481034; doi:10.1371/journal.pntd.0005606)
Supplement: S1 Table — (PDF) [file pntd.0005606.s002.pdf]

S1 Table. Comparison of baseline characteristics between lost to follow-up women and women with complete follow-up.

		Lost to follow-up		Complete follow-up		Total		
			(%)	n	(%)	n		p-value
**Study site**	Colombia	1264	(61.8)	779	(38.1)	2043		**<0.001**
	Guatemala	984	(48.9)	1025	(51)	2009		
	Brazil	797	(48)	860	(52)	1657		
	India	688	(34.7)	1294	(65.2)	1982		
	Papua New Guinea	698	(41.1)	999	(58.8)	1697		
	All countries	4431	(47.2)	4957	(52.8)	9388		
**Age** (years) *		23.3 (5.9) [4410]		23.8 (5.4) [4935]		23.6 (5.6) [9345]		**<0.001**
**Gestational age** (weeks) *		23 (8) [4073]		24(8) [4466]		24 (8) [8539]		**<0.001**
		n	(%)	n	(%)	n	(%)	
**Gestational age** †	1st trimester	562	(13.7)	329	(7.4)	891	(10.4)	**<0.001**
	2nd trimester	1764	(43.3)	1844	(41.2)	3608	(42.2)	
	3rd trimester	1747	(42.8)	2293	(51.3)	4040	(47.3)	
**Weight** (kg) *		57.6 (10.1) [4351]		57.8 (10.1) [4873]		57.7 (10.1) [9224]		0.297
**Height** (cm) *		154.1 (6.1) [4405]		154.1 (6.1) [4923]		154.1 (6.1) [9238]		0.904
**Haemoglobin** (gr/dL)		10.7 (1.8) [4276]		10.3 (1.8) [4783]		10.5 (1.8) [9059]		**<0.001**
		n	(%)	n	(%)	n	(%)	
**Gravidity**	Primigravidae	1733	(39.1)	1986	(40.1)	3719	(39.6)	**0.029**

1 to 3 pregnancies	1707	(38.5)	1973	(39.8)	3680	(39.2)	
4 or > pregnancies	983	(22.2)	989	(19.9)	1972	(21)	
**Overall anaemia at baseline** (Hb<11 g/dl)	2282	(53.3)	2930	(61.2)	5158	(56.9)	**<0.001**
**Severe anaemia at baseline** (Hb<7 g/dL)	117	(2.7)	165	(3.4)	282	(3.1)	**<0.001**
**History of fever last 24h**	82	(1.9)	105	(2.3)	187	(2.1)	0.308
**Fever** (axillary temperature ≥ 37.5 ºC)	47	(1.1)	50	(1)	97	(1)	0.803
**Previous malaria episodes during current pregnancy**	131	(3.2)	161	(3.6)	292	(3.4)	0.146
**Slept under a bednet the night before**	1698	(41.4)	1730	(38.5)	3428	(39.9)	**0.037**
**Indoor residual spraying**	1101	(31.2)	1077	(27.8)	2178	(36.7)	**<0.001**
**Antimalarials taken during pregnancy** (as treatment)	104	(2.5)	145	(3.2)	249	(2.9)	**0.019**
**Taking malaria prophylaxis**	18	(0.5)	27	(0.6)	45	(0.5)	**<0.001**

* Arithmetic Mean (SD) [n].

† 1st trimester: 0-12 weeks, 2nd trimester: 13-24 weeks, 3rd trimester: 25-40 weeks.
