# Supplementary material for: Burden and impact of Plasmodium vivax in pregnancy: A multi-centre prospective observational study
Source: PLoS Negl Trop Dis. 2017 Jun 12;11(6):e0005606. doi: 10.1371/journal.pntd.0005606 (PMC5481034; doi:10.1371/journal.pntd.0005606)
Supplement: S2 Table — (PDF) [file pntd.0005606.s003.pdf]

S2 Table. Incidence of malaria detected through passive case detection (PCD) and through active detection of infection (ADI), overall and by species and country.

	Overall			Colombia			Guatemala			Brazil			India			Papua New Guinea		
	**Events**	**PYAR***	**Rate**	**Events**	**PYAR**	**Rate**	**Events**	**PYAR**	**Rate**	**Events**	**PYAR**	**Rate**	**Events**	**PYAR**	**Rate**	**Events**	**PYAR**	**Rate**
**Detected through PCD†**																		
Incidence of *P. vivax* clinical malaria	63	1659	0.037	19	362	0.052	1	319	0.003	11	308	0.035	23	376	0.060	9	294	0.030
Incidence of *P. falciparum* clinical malaria	49	1685	0.029	8	370	0.021	0	325	0.000	0	314	0.000	1	382	0.002	40	294	0.135
Incidence of any *Plasmodium* clinical malaria	103	1672	0.061	25	368	0.068	1	320	0.003	8	311	0.025	24	376	0.063	45	297	0.151
**Detected through ADI†**																		
Incidence of *P. vivax* infection	109	1680	0.064	18	367	0.049	3	324	0.009	10	313	0.031	23	381	0.060	55	295	0.186
Incidence of *P. falciparum* infection	183	1669	0.109	14	369	0.037	0	325	0.000	0	315	0.000	1	382	0.020	168	278	0.602
Incidence of any *Plasmodium* infection	285	1656	0.172	32	365	0.087	3	324	0.009	10	313	0.031	24	381	0.062	216	273	0.789

* PYAR: person-years at risk.

† PCD: Passive Case Detection at the outpatient clinic. ADI: Active Detection of Infection at the ANC scheduled visits.
